# Conversion surgery after chemoimmunotherapy for esophageal squamous cell carcinoma with gastric intramural metastasis: a case report and the literature review

**DOI:** 10.1093/jscr/rjag073

**Published:** 2026-02-17

**Authors:** Yuki Kitano, Kotaro Sugawara, Yoshiyuki Miwa, Koichi Yagi, Yoshifumi Baba

**Affiliations:** Department of Gastrointestinal Surgery, Graduate School of Medicine, The University of Tokyo, Tokyo, Japan; Department of Gastrointestinal Surgery, Graduate School of Medicine, The University of Tokyo, Tokyo, Japan; Department of Gastrointestinal Surgery, Graduate School of Medicine, The University of Tokyo, Tokyo, Japan; Department of Gastrointestinal Surgery, Graduate School of Medicine, The University of Tokyo, Tokyo, Japan; Department of Gastrointestinal Surgery, Graduate School of Medicine, The University of Tokyo, Tokyo, Japan

**Keywords:** esophageal squamous cell carcinoma, intramural metastasis, immune checkpoint inhibitor, conversion surgery

## Abstract

Immune checkpoint inhibitors (ICIs) have recently emerged as promising first-line treatments. Among stage IVb esophageal squamous cell carcinoma (ESCC) cases, gastric wall metastasis is rare, and conversion surgery after ICI therapy in such cases is extremely uncommon. We report a case of stage IVb ESCC with gastric intramural metastasis (IM) successfully treated with conversion surgery following ICI-based chemoimmunotherapy, along with a review of the relevant literature. A 73-year-old man was diagnosed with ESCC with gastric IM (Mt, SCC, cT3brN1M1b [IM-St], stage IVb). He received three cycles of combination immunochemotherapy with cisplatin, 5-fluorouracil, and pembrolizumab. Conversion surgery was subsequently performed. Pathological examination demonstrated a complete response, with no residual carcinoma in either the esophagus or the gastric wall. The patient remains recurrence-free 8 months postoperatively. Further studies are warranted to clarify the optimal criteria for assessing ICI treatment response and determining indications for conversion surgery in such cases.

## Introduction

Although multimodal therapy for esophageal squamous cell carcinoma (ESCC) has advanced, survival outcomes for patients with initially unresectable (cStage IV, cT4b, and/or cM1b) disease remain dismal [1]. Immune checkpoint inhibitors (ICIs) have recently become part of the standard treatment for ESCC [2, 3]. In Japan, combination chemotherapy with ICIs is now recommended as the standard regimen for stage IVb ESCC. Four first-line options are currently endorsed: CF (cisplatin +5-fluorouracil) plus pembrolizumab, CF plus nivolumab, CF plus tislelizumab, and nivolumab plus ipilimumab [4].

Despite these advances, the median overall survival (OS) of patients with stage IVb ESCC remains 7.6–10.2 months [5]. In selected cases, however, patients responding well to initial therapy and subsequently undergoing curative resection (conversion surgery) have shown favorable outcomes, with reported 3-year survival rates of 32.8%–40% [5–7]. Most previously reported cases underwent neoadjuvant chemotherapy with DCF (docetaxel + cisplatin +5-FU) or CF [8]. More recently, successful conversion surgeries following ICI-based induction therapy have also been described [9].

Gastric wall metastasis from esophageal cancer occurs in only 1.0%–2.7% of cases and carries a poor prognosis. Studies on gastric intramural metastasis from esophageal cancer are summarized in Table 1 [10, 11]. Reports of conversion surgery for ESCC with gastric intramural metastasis (IM) are extremely limited [10]. To our knowledge, none have involved prior ICI-based therapy. Here, we present the first reported case of stage IVb ESCC with gastric IM in which ICI-based chemoimmunotherapy enabled curative conversion surgery.

**Table 1:** Summary of studies on gastric intramural metastasis from esophageal cancer.

**Year**	**Author**	**Ref. No**	**Age (years)**	**Sex**	**Location of gastric lesion**	**Treatment**	**Outcome**
2025	Hao-Ying W *et al*.		76 66	M M	Lesser curvature Lesser curvature	Chemotherapy+ ICI Surgery	Alive without recurrence (6 months) Unknown
2024	Kaneko D *et al*.	10	75	M	Just below GEJ	Chemotherapy+ Surgery	Alive without recurrence (5 years)
2023	Wakasugi A *et al*.	11	72	F	Lesser curvature	Chemotherapy stopped →surgery+ adjuvant Nivolumab	Alive without recurrence (9 months)
2021	Roul PK, *et al*.		37	M	Fundus and Upper body	Chemotherapy	Unknown
2020	Shuto M *et al*.		71	M	Cardia (6 cm)	CRT	Alive without recurrence (10 months)
2018	Hosoda Y *et al*.	12	73	M	Anterior wall of cardia (7 cm)	Surgery	Died (3 months)
2012	Nabeki B *et al*.	13	51	F	Cardia (5.3 cm)	Surgery+ Adjuvant Chemotherapy	LN recurrence (9 months) →Died (29 months)
2012	Kiuchi R *et al*.	14	75	F	Cardia (2 cm)	ESD + CRT → Surgery	Liver metastasis (2.5 years) → Died (3.7 years)
2012	Iwatani S *et al*.		76	M	Upper posterior wall (7 cm)	Chemotherapy	Died 9 months after first visit
2010	Hashimoto N *et al*.	15	86	M	Posterior wall (−)	3D-CRT	Alive without recurrence (17 months)
2008	Hata H *et al*.		62	M	Upper lesser curvature (8 cm)	Surgery	LN recurrence (10 months) → Died (16 months)
2003	Ebihara Y *et al*.		45	M	Cardia (6 cm)	Surgery	Alive without recurrence (9 years)
2003	Shimizu Y *et al*.		72	—	—	Surgery	Alive without recurrence (8 years)

Abbreviations: LN, Lymph node; CRT, chemoradiotherapy; 3D-CRT, three-dimensional conformal radiation therapy; ESD, Endoscopic Submucosal Dissection; ICI, Immune checkpoint inhibitor.

## Case presentation

A 73-year-old man developed dysphagia in August 2024. In November 2024, he was diagnosed at a local clinic with esophageal cancer and referred to our hospital for further evaluation and management. Laboratory tests were unremarkable except for a mildly elevated squamous cell carcinoma antigen level (4.0 ng/mL).

**Figure 1:**
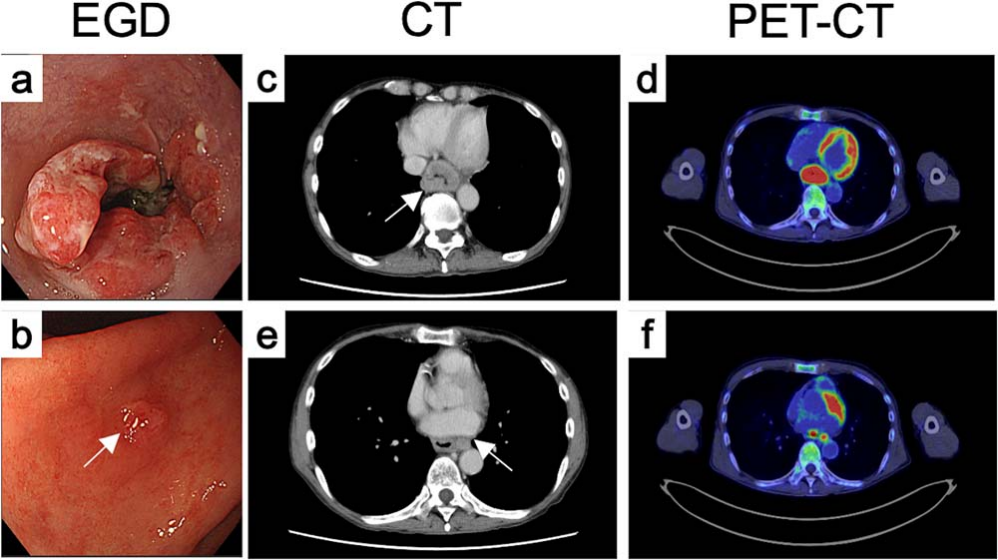
Endoscopic, CT, and FDG-PET findings before treatment. Endoscopic findings of (a) the primary tumor and (b) the gastric IM before treatment. (c) CT and (d) FDG-PET findings of primary tumor before treatment. (e) CT and (f) FDG-PET findings of metastatic LN.

Endoscopy revealed a circumferential type 3 tumor with spontaneous bleeding, located 35–40 cm from the incisors (Fig. 1a). A slightly irregular elevated lesion ~1 cm in size was observed on the lesser curvature of the posterior gastric angle (Fig. 1b). Computed tomography (CT) demonstrated circumferential thickening of the lower esophagus with suspected extramural invasion (Fig. 1c) and enlarged regional lymph nodes near the aorta (Fig. 1e). Positron emission tomography (PET)-CT showed 18F-fluorodeoxyglucose (FDG) uptake corresponding to these lesions (Fig. 1d and f), but no abnormal uptake in the gastric wall.

**Figure 2:**
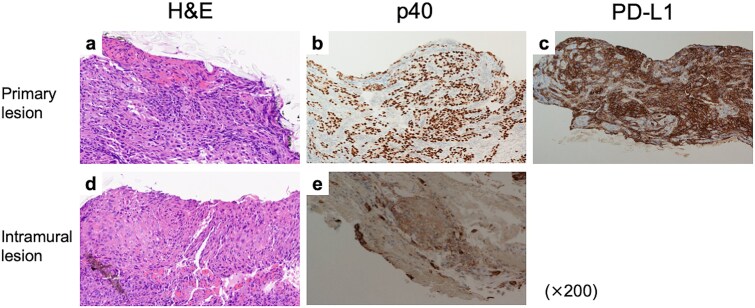
Pathological findings of tumors before treatment. (a) H&E stain, (b) p40 IHC, and (c) PD-L1 IHC findings of the primary tumor. CPS was estimated as 90. (d) H&E stain and (e) p40 IHC of the gastric IM.

Histologically, hematoxylin and eosin (H&E) staining revealed atypical squamous cells forming nests and cords (Fig. 2a), positive for p40 (Fig. 2b), confirming squamous cell carcinoma. Programmed death-ligand 1 (PD-L1) expression was high, with a combined positive score (CPS) of 90 (Fig. 2c). The gastric lesion showed atypical squamous cells with focal keratinization, consistent with intramural metastasis (Fig. 2d and e).

**Figure 3:**
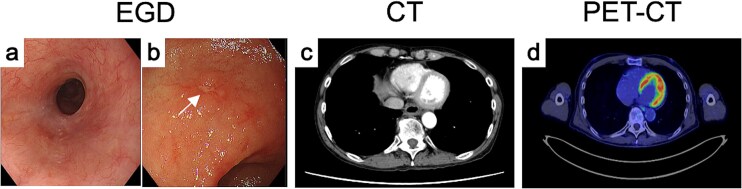
Endoscopic, CT, and FDG-PET findings after treatment. After induction chemoimmunotherapy, both (a) the primary tumor and the (b) IM markedly shrank. (c) CT and (d) FDG-PET findings of primary tumor after treatment.

The final diagnosis was advanced, unresectable ESCC of the middle thoracic esophagus (Mt, SCC, cT3brN1M1b [IM-St], stage IVb). The patient received three cycles of CF plus pembrolizumab. Post-treatment evaluation showed fibrotic scarring at both sites (Fig. 3a and (b)), and biopsies revealed no residual tumor. CT and PET-CT demonstrated marked regression and no abnormal FDG uptake (Fig. 3c and d). The overall response was judged as a partial response, and conversion surgery was planned.

In March 2025, the patient underwent thoracoscopic subtotal esophagectomy with a cervical incision and laparoscopic abdominal approach, with two-field lymph node dissection. Reconstruction was achieved using a retrosternal gastric conduit and cervical esophagogastrostomy. A feeding jejunostomy was also placed. Postoperatively, the patient developed aspiration pneumonia (Clavien–Dindo grade IIIa) and anastomotic leakage (grade II), both managed conservatively. He was discharged on postoperative day 43.

**Figure 4:**
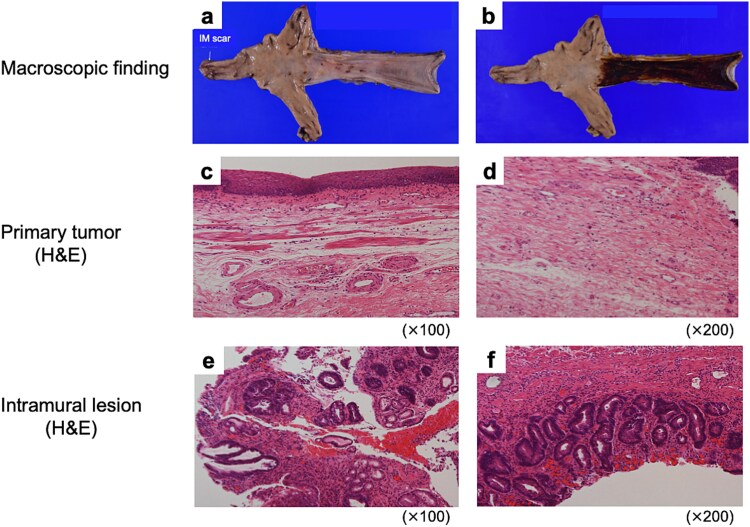
Pathological findings of resected specimens. Macroscopic findings of (a) non-stained and (b) iodine-stained resected specimen. H&E stain findings of the lesions (c, d) where the primary tumor was originally located, and (e, f) where the gastric IM was originally present.

Macroscopically, no gross tumor was evident (Fig. 4a). Iodine staining revealed no unstained area (Fig. 4b). Histological examination showed complete pathological response, with no viable tumor cells in either the esophageal or gastric lesions (Fig. 4c–f). The patient remains recurrence-free 8 months after surgery.

## Discussion

We report a rare case of initially unresectable metastatic ESCC achieving a complete response following multimodal therapy including ICI-based chemoimmunotherapy, which enabled curative conversion surgery.

ICIs have become integral to ESCC management, increasing opportunities for conversion surgery. Several studies have reported conversion surgeries after induction chemotherapy for stage IVb ESCC [5–7]. In one retrospective series of 71 patients treated with ICI-based therapy (March 2021–December 2022), the disease control rate was 80%, median progression-free survival 9.7 months, and the conversion surgery rate 7%. Grade ≥ 3 immune-related adverse events occurred in 17%, consistent with previous trials [12].

Intramural metastasis occurs in 1.0%–4.7% of ESCC cases and is associated with poor prognosis, with median survival of 11 months in resected and 4 months in unresected cases [13]. Even when technically resectable, gastric IM is classified as M1b (distant metastasis) in the Japanese Classification of Esophageal Cancer. Currently, there is no established treatment strategy for esophageal IM. Preoperative CF chemotherapy has limited efficacy, and regimens such as DCF or ICI-based therapy are increasingly used [14]. Although one case of conversion surgery after induction chemotherapy for ESCC with gastric IM has been reported [10], only a single case following ICI-based therapy has been described, and it was performed with palliative rather than curative intent [11]. Thus, further accumulation of clinical experience is necessary.

Whether surgical resection is warranted in patients showing excellent response remains debatable. The complete response rate to induction chemotherapy for stage IVb ESCC is only ~3% [5]. Because preoperative confirmation of complete response is difficult [7], curative resection may be justified in cases demonstrating strong response to systemic therapy. Conversely, active surveillance strategies have shown promise, particularly after chemoradiotherapy [15]. Given the potent efficacy of ICI-based regimens, determining whether surgery remains necessary in such responders is a key future challenge.

## Conclusion

We describe a rare case of ESCC with gastric intramural metastasis successfully treated with conversion surgery following ICI-based chemoimmunotherapy. ICI-based chemoimmunotherapy achieved a complete response for the patient with the far-advanced ESCC. Further research is required to establish optimal methods for evaluating treatment response and defining surgical indications after ICI therapy.

## Data Availability

Data sharing is not applicable to this article, as no datasets were generated or analyzed during the current study.
